# A Zero-Cross Detection Algorithm for Cavity-Length Interrogation of Fiber-Optic Fabry–Perot Sensors

**DOI:** 10.3390/s19183868

**Published:** 2019-09-07

**Authors:** Zhibo Ma, Zechen Song, Xirui Huang, Tongxin Guo, Weizheng Yuan, Haibin Chen, Tianyang Zhang, Wei Wang

**Affiliations:** 1 Key Lab of Micro/Nano Systems for Aerospace, Ministry of Education, Northwestern Polytechnical University, Xi’an 710072, China; 2 Shaanxi Key Lab of MEMS/NEMS, Northwestern Polytechnical University, Xi’an 710072, China; 3 School of Optoelectronic Engineering, Xi’an Technological University, Xi’an 710021, China; 4 Shaanxi Province Key Lab of Photoelectric Measurement and Instrument Technology, Xi’an Technological University, Xi’an 710021, China

**Keywords:** fiber-optic sensor, Fabry–Perot cavity, peak-to-peak method, zero-cross detection

## Abstract

A zero-cross detection algorithm was proposed for the cavity-length interrogation of fiber-optic Fabry–Perot (FP) sensors. The method can avoid the inaccuracy of peak determination in the conventional peak-to-peak method for the cavity-length interrogation of fiber-optic FP sensors caused by the slow variation of the spectral power density in peak neighboring regions. Both simulations and experiments were carried out to investigate the feasibility and performance of the zero-cross detection algorithm. Fiber-optic FP sensors with cavity lengths in the range of 150–1000 μm were successfully interrogated with a maximum error of 0.083 μm.

## 1. Introduction

Fiber-optic sensors, utilizing the modulation effect of external parameters on light intensity, phase, frequency, or polarization to achieve the sensing of various different quantities, have found broad applications in industrial, research, and defense areas because of their advantages of simple structure, compact size, high resolution, chemical passivity, and electromagnetic immunity [1,2,3]. The fiber-optic Fabry–Perot (FP) sensor is a very important type of sensor that can be used for the measurement of many important physical parameters, such as temperature [4,5,6], pressure [7,8], vibration [9], strain/stress [10], and refractive index [11,12].

For real applications of fiber-optic FP sensors, interrogation is one of the most important considerations. Many different methods have been proposed, which can be mainly classified as intensity demodulation and phase demodulation methods. Intensity demodulation is relatively simple and economical. However, it is considerably affected by the stability of the light source, and the measurement results commonly contain relatively large errors [13]. Spectral interrogation is most commonly used for phase demodulation. By illuminating the fiber-optic FP sensors with a wide band source, and receiving and analyzing the reflection spectrum, the cavity length can be obtained. Spectral interrogation can be further classified into the Fourier transformation method [14,15,16,17], cross-correlation method [18,19,20], and peak-to-peak method [21,22,23,24].

The Fourier transform method uses a Fourier or fast Fourier transform procedure to transform the reflection spectrum from the wavelength domain to the cavity-length domain, in which the peak position is determined to be the cavity length. Although the absolute cavity length can be obtained, the resolution is usually limited by the spectral width of the light source.

The cross-correlation method involves cross-correlating the reflection spectrum with a template spectral function of a virtual FP sensor with a tunable cavity length, and determining the maximum of the cross-correlation coefficient to get the real cavity length. The method can yield the cavity length with much higher resolution than those of other methods; however, it is considerably limited by the spectral width of the light source used. If the spectral width of the light source is not wide enough, the accurate extraction of the cavity length cannot be guaranteed, and a large error, up to half of the central wavelength, may be introduced.

The peak-to-peak method locates the wavelength positions of two peaks in the reflection or transmission spectrum and uses them to calculate the cavity lengths of fiber-optic FP sensors. This method can obtain the cavity length in a relatively direct manner. However, near the peaks, especially for a low-finesse FP cavity, the signal changes relatively slowly; as a result, precisely locating the peak position is very difficult, and the cavity-length interrogation resolution is not very high. Using high-finesse FP sensors to sharpen the reflection peaks, which can reduce the difficulty of accurate peak determination, has been proposed; however, sensor fabrication in this scenario is much more complicated [23]. The algorithm can also be improved to increase the interrogation resolution; however, the improvement is very limited, especially for low-finesse FP sensors [24].

To avoid the inaccuracy problem of the peak-to-peak method, a zero-cross detection algorithm for cavity-length interrogation of fiber-optic FP sensors was proposed. The reflection spectrum is transformed from the wavelength domain to optical frequency domain, the equi-period spectral signal is filtered by a raised-cosine function, and two different zero points are selected to obtain the period value, yielding the cavity length. The zero points have the largest slope in the nearby region, thus they can be more easily located precisely than the peak points. Both simulations and experiments were carried out to investigate the feasibility and performance of the proposed algorithm, which was demonstrated to provide a higher resolution than the conventional peak-to-peak method.

## 2. The Principle of Cavity-Length Interrogation

**Figure 1:**
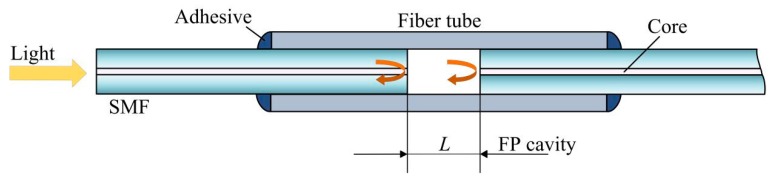
Typical structure of a fiber-optic Fabry–Perot FP sensor, in which *L* is the cavity length.

A fiber-optic FP sensor, whether intrinsic or extrinsic, can be commonly viewed as an optical resonator formed by two parallel reflective surfaces. Figure 1 illustrates the typical structure of a simple fiber-optic FP sensor, formed by a short capillary with two vertically cut single mode fibers (SMFs) inserted in it. The two facets of the two SMFs formed the FP cavity.

Assuming the reflection ratios of the two facets are $R_{1}$ and $R_{2}$, the material filled in the cavity has a reflection index of n, and is lossless. If the cavity length is L, for a wideband incident light with a spectral power density of $I_{0}\left(\lambda \right)$, the reflected light has a spectral distribution [25] of

(1) $$I_{r}\left(\lambda \right)=\frac{R_{1}+R_{2}-2\sqrt{R_{1}R_{2}}\cos\frac{4\pi nL}{\lambda }}{1+R_{1}R_{2}-2\sqrt{R_{1}R_{2}}\cos\frac{4\pi nL}{\lambda }}I_{0}\left(\lambda \right).$$

It can be transformed into the optical frequency domain by ν=c/λ as

(2) $$I_{r}\left(\nu \right)=\frac{R_{1}+R_{2}-2\sqrt{R_{1}R_{2}}\cos\frac{4\pi nLv}{c}}{1+R_{1}R_{2}-2\sqrt{R_{1}R_{2}}\cos\frac{4\pi nLv}{c}}I_{0}\left(\nu \right)$$

where ν is the optical frequency.

**Figure 2:**
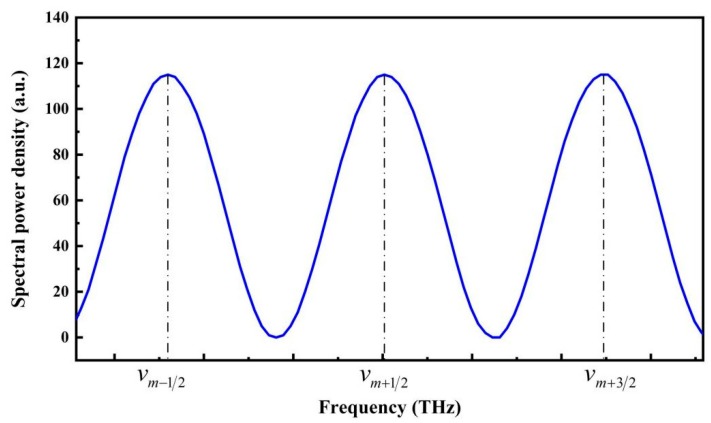
Typical reflection spectrum of a fiber-optic FP sensor in the optical frequency domain, in which, $v_{m-1/2}$, $v_{m+1/2}$, and $v_{m+3/2}$ represent the optical frequencies of three successive reflection peaks, and *m* is an integer related with the orders of the reflection peaks.

It can be seen that, in the optical frequency domain, if the incident light has a uniform spectral power density $I_{0}$, the reflection spectrum of the fiber-optic FP sensor is like that shown in Figure 2, which is a periodic function of the optical frequency, where

(3) $$\frac{4\pi nL}{\lambda_{m+1/2}}=\frac{4\pi nLv_{m+1/2}}{c}=\left(m+\frac{1}{2}\right)2\pi ,\;m=0,1,2,\cdots$$

The *m*-th order peak of the power density of the reflection spectrum is given by

(4) $$v_{m+1/2}=\left(m+\frac{1}{2}\right)\frac{c}{2nL}.$$

In the optical frequency domain, the reflection spectrum of the fiber-optic FP sensor is an equi-period signal, in which the period is the optical frequency difference of the two neighboring peaks; i.e.,

(5) $$T=v_{m+3/2}-v_{m+1/2}=\frac{c}{2nL}.$$

Thus, the cavity length can be expressed as

(6) $$L=\frac{c}{2nT}.$$

However, near the reflection peaks, particularly when the fiber-optic FP sensor is of the low-finesse type, the spectral power density changes relatively slowly with optical wavelength or frequency. Furthermore, under real conditions, noise introduced by various environmental conditions cannot be avoided. As a result, the peak positions cannot be precisely determined. Thus, obtaining the cavity length by calculating the signal period in the frequency domain through the peak positioning of the optical wavelength or frequency commonly involves a large interrogation deviation.

If the fiber-optic FP sensor is a low-finesse type, i.e., $R_{1}<<1$, $R_{2}<<1$, Equation (2) can be approximately given by

(7) $$I_{r}\left(v\right)=A-B\cos\left(\frac{4\pi nL}{c}v\right),$$

where, $A=\left(R_{1}+R_{2}\right)I_{0}$ and $B=2\sqrt{R_{1}R_{2}}I_{0}$. The slope against the optical frequency is

(8) $$\frac{dI_{r}\left(v\right)}{dv}=-\frac{d}{dv}B\cos\left(\frac{4\pi nL}{c}v\right)=B\frac{4\pi nL}{c}\sin\left(\frac{4\pi nL}{c}v\right).$$

It can be noticed that, near optical frequencies

(9) $$v_{m+1/4}=\left(m+\frac{1}{4}\right)\frac{c}{2nL},m=0,1,2,\ldots ,$$

(10) $$v_{m+3/4}=\left(m+\frac{3}{4}\right)\frac{c}{2nL},m=0,1,2,\ldots ,$$

the spectral power density of the reflection light has the largest slope; i.e., the signal changes the fastest in the neighboring regions. Points with the largest slope are often called Q-points. If these Q-points can be precisely determined, then

(11) $$v_{m+5/4}-v_{m+1/4}=v_{m+7/4}-v_{m+3/4}=\frac{c}{2nL}=T.$$

The period of the reflection spectrum in the frequency domain can be written as

(12) $$T=v_{m+5/4}-v_{m+1/4}=v_{m+7/4}-v_{m+3/4}.$$

Thus, the cavity length of the low-finesse fiber-optic FP sensor can be determined through Equation (6). It should be noted that, if the fiber-optic FP sensor is not a low-finesse type, the frequencies of the Q-points will deviate from the positions given by Equation (9) or Equation (10). However, the frequency separation of any two neighboring Q-points with the same sign of the largest slope will still satisfy the condition c/(2nL)=T, thus can be used for the cavity length extraction.

From the viewpoint of Fourier analysis and signal processing, the reflection spectrum contains a constant term, and many cosine oscillating components with different frequencies. At Q-points, the spectral power density values of the reflection spectrum will all equal to the constant term. However, for different fiber-optic FP sensors, different power levels of illuminating light, or different environments, will fluctuate continuously. If the constant term can be eliminated or filtered out, the spectral power density of the reflection spectrum at these Q-points will be zero, which can be more easily positioned under versatile conditions. The Q-points after DC filtering can be called zero-points. By using the zero-cross detection method commonly used in electric signal processing [26], the positions of two Q-points can be precisely located to extract the cavity length of fiber-optic FP sensors.

## 3. Zero-Cross Detection Algorithm

**Figure 3:**
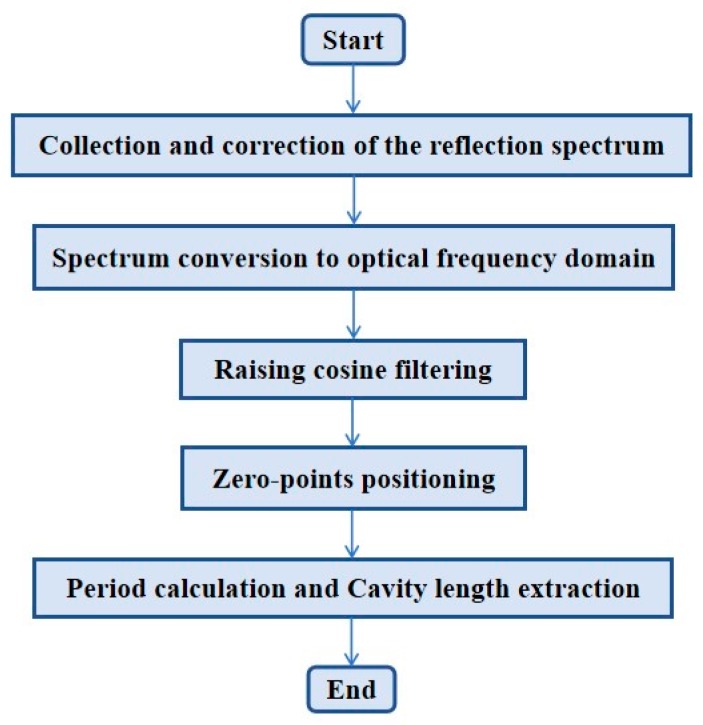
Flowchart of the zero-cross detection algorithm for cavity-length interrogation of fiber-optic FP sensors.

According to the reflection spectral characteristics of fiber-optic FP sensors and the theory of zero-cross detection given above, a zero-cross detection algorithm was proposed, which can avoid the positioning uncertainty in conventional peak-to-peak detection. A flow chart of the algorithm is given in Figure 3. The main procedure of the algorithm is as follows:

First, the reflection spectrum is obtained from an optical spectrum analyzer (OSA), which is then corrected by the output spectrum of the wideband light source to eliminate the nonuniform distribution of its spectral power density. Afterwards, the corrected reflection spectrum is transformed from the wavelength domain to the optical frequency domain. Considering the charge-coupled device (CCD) array used in the OSA, the spectral data is commonly expressed in discrete form as

(13) $$I_{r}\left(\nu_{i}\right)=\frac{R_{1}+R_{2}-2\sqrt{R_{1}R_{2}}\cos\frac{4\pi nLv_{i}}{c}}{1+R_{1}+R_{2}-2\sqrt{R_{1}R_{2}}\cos\frac{4\pi nLv_{i}}{c}}I_{0},i=1,2,3,\ldots$$

where $\nu_{i}$ is the optical frequency of the *i*-th sampling point of the reflection spectrum obtained by the OSA.

Next, the raised-cosine function is constructed

(14) $$C\left(j\right)=-\frac{2}{M+1}\cos\left(\frac{2\pi j}{M+1}\right),j=0,1,\ldots ,M,$$

For the j-th point in a period, and M+1 is the number of total points in one full period of the raised-cosine function. When M+1 is equal or nearly equal to the total of sampling points of the reflection spectrum in one period, the raised-cosine filtering will reach the optimal effect. By roughly estimating the period of the reflection spectrum in the optical frequency domain and the number of sampling points in one period, the value of M+1 can be given. The raised-cosine function is considered for correlation with the reflection spectrum in the optical frequency domain as

(15) $$Y\left(v_{k}\right)=-\frac{2}{M+1}\sum_{j=k}^{M+k}\cos\left(\frac{2\pi j}{M+1}\right)I\left(v_{j}\right),\;k=0,1,2,\ldots$$

Through the raised-cosine filtering, the constant term can be filtered out without affecting the period of the reflection spectrum in the frequency domain.

Next, two zero points in two ascending (or descending) segments of the filtered spectral signal are located. To determine each zero point, the nearest point above zero and the nearest point below zero are found; then, these points are used to obtain the precise zero point by a linear fitting method. Assuming the nearest point above and below zero are the k-st and (k+1)st points, respectively; their coordinates are $\left(v\left(k\right),Y\left(v_{k}\right)\right)$ and $\left(v\left(k+1\right),Y\left(v_{k+1}\right)\right)$, respectively. By linear fitting, the slope coefficient can be written as

(16) $$a=\frac{Y\left(v_{k+1}\right)-Y\left(v_{k}\right)}{v\left(k+1\right)-v\left(k\right)},$$

and the intercept b is

(17) $$b=Y\left(v_{k}\right)-\frac{Y\left(v_{k+1}\right)-Y\left(v_{k}\right)}{v\left(k+1\right)-v\left(k\right)}v\left(k\right).$$

Thus, we have a linear equation

(18) y=av+b

From the equation, the precise frequency corresponding to the zero point is

(19) $$v=-\frac{b}{a}$$

Finally, the period is calculated, and the cavity length is extracted. Denote frequencies of the two zero points as $v_{I}$ and $v_{\mathrm{II}}$; suppose the number of periods between them is N. Then, the period is calculated by

(20) $$T=\frac{v_{II}-v_{I}}{N},$$

and the cavity length is extracted through Equation (6).

## 4. Numerical Simulation

**Figure 4:**
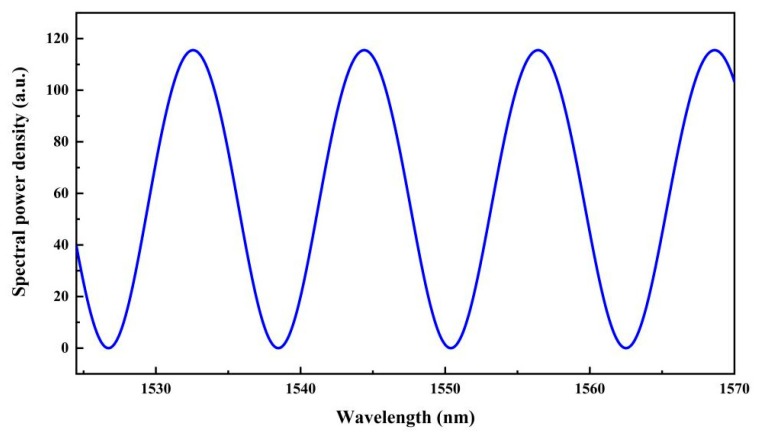
Simulated reflection spectrum of a fiber-optic FP sensor with a cavity length of 100 μm.

The feasibility and performance of the interrogation algorithm was first investigated through a numerical simulation. An air-gap fiber-optic FP sensor with a cavity length of 100 µm was simulated, and its reflection spectrum in the wavelength range of 1524–1570 nm was calculated, as shown in Figure 4. The considered spectral range was the same as the amplified spontaneous emission (ASE) source we used in the experiment.

**Figure 5:**
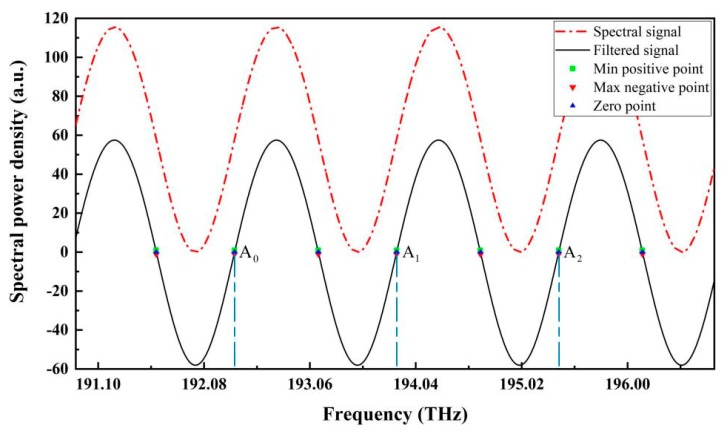
Simulated reflection spectrum in the optical frequency domain, in which A_0_, A_1_, and A_2_ are three zero-cross points in ascending segments of the filtered spectral signal.

According to the zero-cross detection algorithm described previously, the simulated spectrum was first transformed into the optical frequency domain; then, it was filtered through the raised-cosine filtering method. The spectral signal after filtering was in oscillating form without the constant term. All the zero-cross points in the considered range were located by linear fitting using the positive and negative points nearest to zero in the filtered spectral signal, as shown in Figure 5. In the figure, the fitted zero points, and the nearest positive and negative points are all denoted.

The frequencies of the three zero points A_0_, A_1_, and A_2_ were 192.3711548 THz, 193.8711248 THz, and 195.3711248 THz, respectively. When A_0_ and A_1_ in two neighboring ascending segments of the filtered spectral signal were selected, the period *T* of the spectral signal in the optical frequency domain was calculated to be 1.49997 THz. According to Equation 6, the cavity length was calculated to be 100.002 μm, which deviated from the standard value of 0.002 μm, with a relative error of approximately 0.002%. When using the first and last zero-points A_0_ and A_2_, to calculate the cavity length, two full periods can be covered; the calculated cavity length was 100.001 μm, which deviated from the standard value of 0.001 μm. Evidently, when *N* periods can be covered, the cavity length’s calculating error can be further reduced about *N* times. Therefore, in real cavity length interrogation through the zero-cross detection algorithm, it is better to directly position the first and last zero points for the calculation.

**Figure 6:**
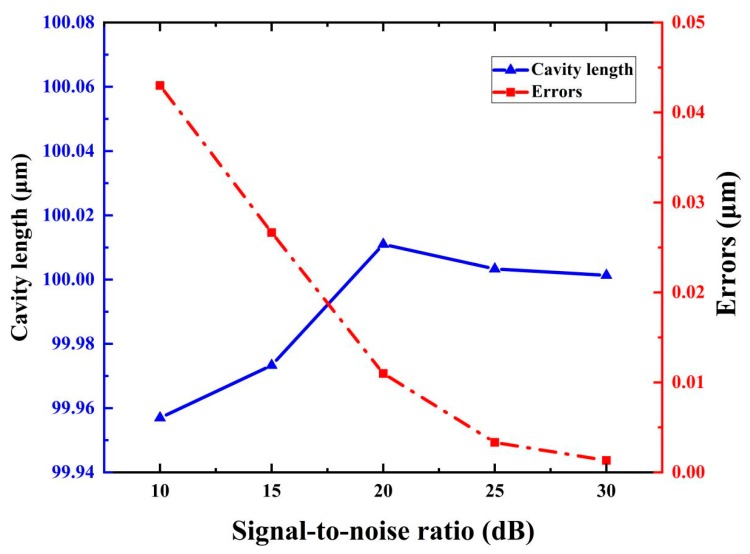
Simulated interrogation results and errors of cavity length under different SNR conditions. In the zero-points selection, two periods were covered.

The cavity lengths for different noise levels were also determined, as shown in Figure 6. The simulated interrogation results for the 100 μm FP cavity under different noise levels were also obtained, which are shown in Figure 6. It can be seen that, when the signal-to-noise ratio (SNR) is 30 dB, the error is approximately 0.001 μm. Even when the SNR reached a level of 10 dB, the error was not larger than 0.043 μm. Thus, the method has a good resistance to noise.

**Figure 7:**
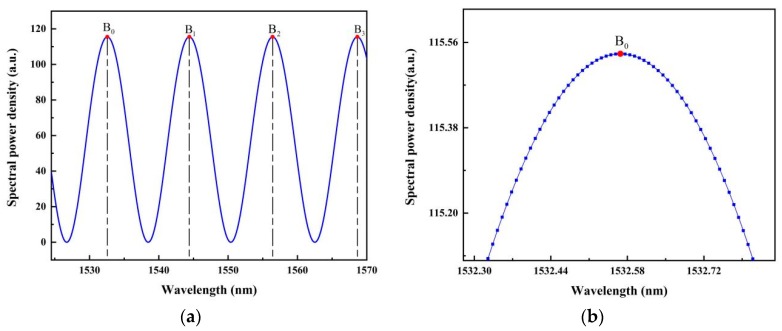
(**a**) Calculation of the cavity length using two neighboring apexes based on the peak-to-peak method; (**b**) enlarged view of the reflection spectrum at the wavelength position of one peak. B_0_, B_1_, B_2_, and B_3_ are four reflection peaks.

For comparison, the same simulated reflection spectrum was also treated through the peak-to-peak method; as seen in Figure 7a, four peaks can be found. Wavelengths of the four peaks B_0_, B_1_, B_2_, and B_3_ were positioned to be 1532.57041 nm, 1544.12235 nm, 1556.41714 nm, and 1568.59718 nm, respectively. When two neighboring peaks, B_0_ and B_1_, were selected directly in the wavelength domain, through the conventional cavity length calculating formula $L=\lambda_{0}\lambda_{1}/2(\lambda_{1}-\lambda_{0})$, the interrogation yielded a length of 100.243 μm, which deviated from the predetermined cavity length by 0.243 μm. When the first and last peaks B_0_ and B_3_ were selected to calculate the cavity length, the result was 100.092 μm, with an error of 0.092 μm. Although in the peak-to-peak method, by the consideration of multiple peaks, the cavity length error can be effectively reduced, in comparison, the result given by the zero-cross detection was much better. The local enlargement of the spectrum near point B_0_ is shown in Figure 7b. Clearly, the spectral power densities of the points near the peak were very close, and the real peak position in this situation is difficult to locate. On the contrary, near the zero points, the data have the largest slopes, which can be determined with high precision; that is why the new proposed zero-cross detection algorithm yields much better interrogation resolution.

**Figure 8:**
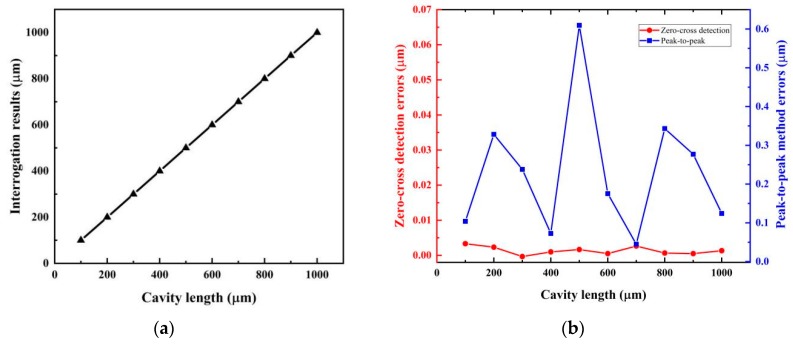
(**a**) Simulated cavity length interrogation results for 10 different FP cavity lengths in the range 100–1000 μm under a signal-to-noise ratio (SNR) of 20 dB; (**b**) cavity length calculating errors acquired by the zero-cross detection and peak-to-peak methods.

In Figure 8, we also give out the simulation results for 10 different FP cavity lengths in a range 100–1000 μm under the same SNR level of 20 dB. Both the calculated cavity lengths and their errors are demonstrated. In the calculation, the first and last zero points in ascending segments were used. It was found that the results all fitted the predetermined values well; the worst cavity length errors were less than 0.003 μm. For comparison, the cavity length calculating errors by the peak-to-peak method were also given. Evidently, the zero-cross detection algorithm has a better resolution in the full range.

## 5. Experimental Verification

**Figure 9:**
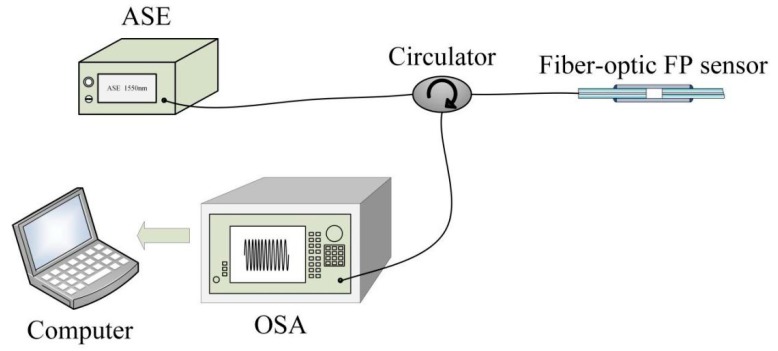
Schematic of the experimental system for cavity-length interrogation.

**Figure 10:**
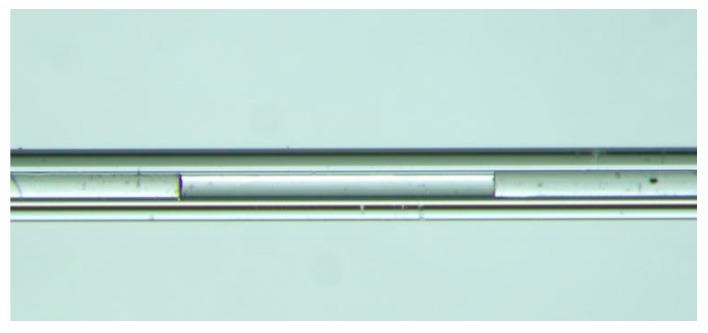
Microscopic photograph of an air-gap fiber-optic FP sensor to be interrogated.

To investigate the performance of the zero-cross detection algorithm for cavity-length interrogation, an experimental interrogation system composed of an ASE source, an optical circulator, an OSA, and a computer was constructed. A schematic diagram of the system is shown in Figure 9. Several air-gap fiber-optic FP sensors with different cavity lengths were fabricated and connected to the interrogation system for cavity extraction. One of the fabricated air-gap fiber-optic FP sensors is shown in Figure 10. The ASE source emitted a broadband light with a center wavelength of 1550 nm, and a 3-dB spectral bandwidth of 46 nm. The light was coupled to the fiber-optic FP sensor through the optical circulator. The reflected light was returned back to the circulator again and finally received by the OSA to yield the reflection spectrum in the optical wavelength domain. Finally, the reflection spectrum was sent to the computer to interrogate the cavity length of the fiber-optic FP sensor through a program written according to the proposed zero-cross algorithm.

**Figure 11:**
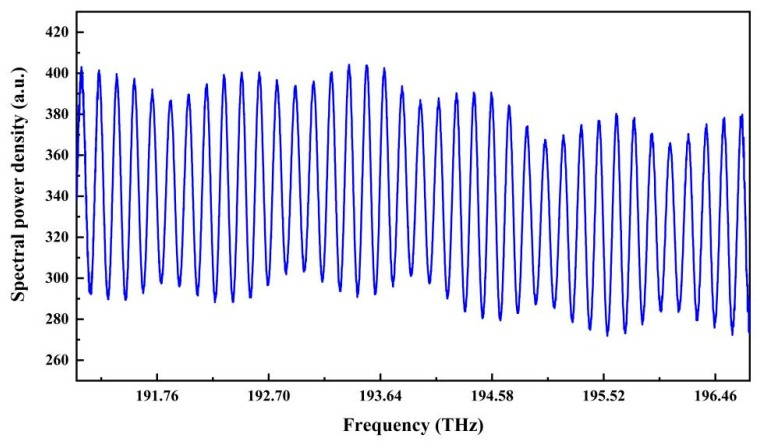
The reflection spectrum in the optical frequency domain of a fiber-optic FP sensor with a cavity length of 999.482 μm.

The reflection spectrum for a cavity length of 999.482 μm is shown in Figure 11, which has multiple peaks in the spectrum.

**Figure 12:**
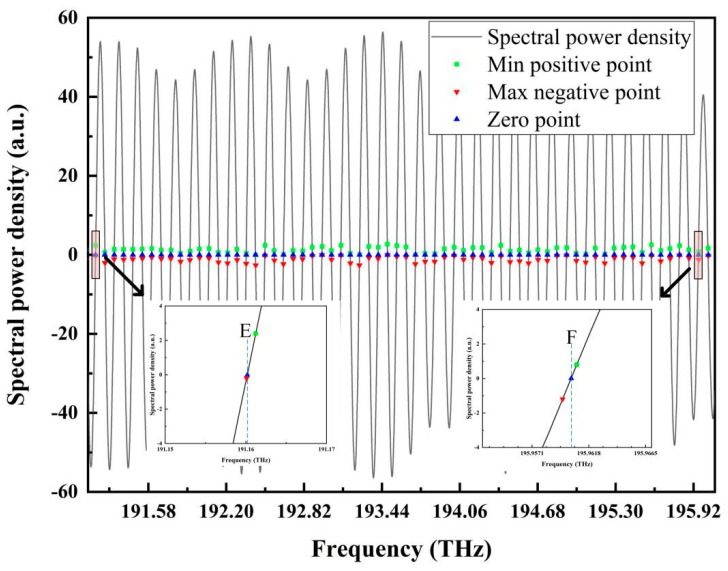
Raised-cosine filtering and zero-cross point positioning. Local enlargements of the first and last zero points in ascending segments are also given, in which the two zero points are marked by E and F.

The reflection spectrum was first filtered by raised-cosine filtering; then, the zero-cross points were found, as shown in Figure 12, in which the local enlargement of the first and last zero-cross points in ascending segments are given.

From the two zero points E and F, which had frequencies of 191.1601443 THz and 195.9625251 THz, respectively, the period of the reflection signal in the optical frequency domain can be calculated to be 0.1500744 THz. Then, the cavity length was calculated as 999.504 μm, which deviated from the standard cavity length of 999.482 μm by 0.022 μm.

By using the same method, 16 other fiber-optic FP sensors with different standard cavity lengths ranging from 150 μm to 1000 μm were also interrogated; the results and errors are listed in Table 1. Compared with the simulation results, all of the cavity lengths’ interrogating errors got relatively larger; many increased 10 times, which may have been caused by the drift of the source spectrum, environment vibrations, or circuit noises. In the table, the results and errors for the peak-to-peak method are also given. It was found that the largest error for the zero-cross detection method was 0.083 μm, which is far less than the largest error, 0.874 μm, of the peak-to-peak method.

The standard cavity lengths were given by the cross-correlation method [19,20]. A white-light cross-correlation measurement system was built by the using of a wide band superluminescent diode (SLD) (3-dB bandwidth of 90 nm) as the light source, and a high resolution OSA with a wavelength resolution of 30 pm to get the reflection spectrum. Based on the reflection spectrum, and using the cross-correlation method to extract the cavity length, sub-nanometer-level resolution can be acquired.

**Figure 13:**
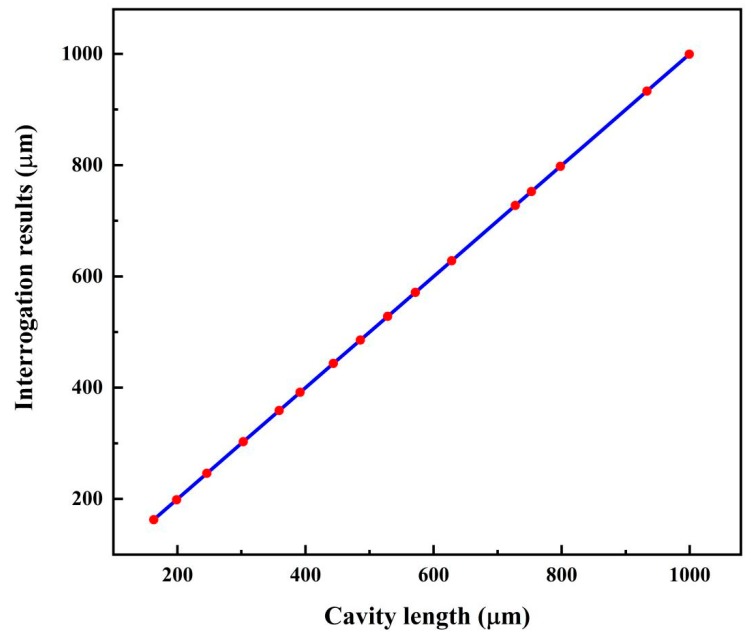
Cavity length interrogation results for fiber-optic FP sensors with different cavity lengths in the range of 150–1000 μm.

The relation between the standard cavity length and the zero-cross detection is shown in Figure 13, with a correlation coefficient of 0.99994. From the experiment, it can be concluded that the zero-cross detection algorithm can be used for the cavity-length interrogation of fiber-optic FP sensors, and the interrogation resolution is far better than that of the peak-to-peak method.

In real applications, we always have a concern about the computational requirements. Compared with the peak-to-peak method, only two more significant steps were needed: one is the DC filtering and one is the zero interpolation. Whether it is the peak-to-peak method, or zero-cross detection method, the calculation toll is not too high; the zero-cross detection method only increases a limited number of calculations. Actually, the bottleneck on the interrogation rate is the frame rate of the CCD used in the OSA. Most OSAs can only work below 1 kHz. For the high-speed OSA module made by Ibsen Photonics, working with a frame rate of about 20 kHz, when using FPGA or DSP as the processing chip, both the peak-to-peak method and the zero-cross detection method can fulfill their work in time.

## 6. Conclusions

Based on the basic characteristics of the reflection spectrum of fiber-optic FP sensors in the optical frequency domain, a zero-cross detection algorithm was proposed and investigated for cavity-length interrogation. For the large slope of the signal near the zero point, the period of the interference signal in the frequency domain can be determined with high precision; as a result, the cavity-length interrogation resolution can be greatly improved compared with the conventional peak-to-peak method. Simulations showed that for a fiber-optic FP sensor with a cavity length in the range of 100 to 1000 μm, the largest cavity length error was less than 0.003 μm. Interrogation experiments for different cavity lengths in the range of 150 to 1000 μm showed the worst error of 0.083 μm, which is much better than the results of the peak-to-peak method. With the proposed method, the cavity length of the fiber-optic FP sensor could be obtained with high resolution in a relatively direct way, which can be applied to various fiber-optic FP sensing systems.

## Figures and Tables

**Table 1:** Interrogation results and errors for fiber-optic FP sensors with different cavity lengths. Both results obtained by the zero-cross detection algorithm and the peak-to-peak method are given for comparison. The column ZC Sim gives the cavity lengths given by zero-cross simulation; ZC Exp and P2P Exp, respectively, give the cavity lengths acquired from the experimental spectra; the columns Error ZCs, Error ZCe, and Error P2Pe are the corresponding cavity lengths interrogating the errors.

Standard Cavity Length/μm	ZC Sim/μm	Error ZCs/μm	ZC Exp/μm	Error ZCe/μm	P2P Exp/μm	Error P2Pe/μm
163.008	163.005	0.003	162.925	0.083	162.774	0.234
198.555	198.556	0.001	198.506	0.049	199.217	0.662
246.067	246.063	0.004	246.058	0.009	246.649	0.582
303.009	303.011	0.002	303.002	0.007	303.244	0.235
359.047	359.05	0.003	358.984	0.063	358.734	0.313
391.67	391.672	0.002	391.684	0.014	392.139	0.469
443.502	443.505	0.003	443.547	0.045	443.641	0.139
485.794	485.793	0.001	485.761	0.033	485.879	0.085
528.283	528.282	0.001	528.331	0.048	528.609	0.326
571.425	571.423	0.002	571.420	0.005	571.803	0.378
628.191	628.192	0.001	628.168	0.023	628.625	0.434
727.549	727.546	0.003	727.524	0.025	727.781	0.232
752.681	752.684	0.003	752.676	0.005	752.628	0.053
798.031	798.03	0.001	798.075	0.044	797.157	0.874
933.077	933.076	0.001	933.085	0.008	933.411	0.334
999.482	999.483	0.001	999.504	0.022	999.858	0.376

## References

[1] Ohno H., Naruse H., Kihara M., Shimada A. (2001). Industrial Applications of the BOTDR Optical Fiber Strain Sensor. Opt. Fiber Technol..

[2] Consales M., Buosciolo A., Cutolo A., Breglio G., Irace A., Buontempo S., Petagna P., Giordano M., Cusano A. (2011). Fiber optic humidity sensors for high-energy physics applications at CERN. Sens. Actuators B Chem..

[3] Bucaro J.A., Dardy H.D., Carome E.F. (1977). Fiber-optic hydrophone. J. Acoust. Soc. Am..

[4] Lee C.E., Taylor H.F. (1991). Fiber-optic Fabry-Perot temperature sensor using a low-coherence light source. J. Lightwave Technol..

[5] Zhu Y., Huang Z., Shen F., Wang A. (2005). Sapphire-fiber-based white-light interferometric sensor for high-temperature measurements. Opt. Lett..

[6] Jedrzejewska-Szczerska M., Wierzba P., Chaaya A.A., Bechelany M., Miele P., Viter R., Mazikowski A., Karpienko K., Wróbel M. (2015). ALD thin ZnO layer as an active medium in a fiber-optic Fabry–Perot interferometer. Sens. Actuators A-Phys..

[7] Kao T.W., Taylor H.F. (1996). High-sensitivity intrinsic fiber-optic Fabry-Perot pressure sensor. Opt. Lett.

[8] Yi J., Lally E., Wang A., Xu Y. (2010). Demonstration of an All-Sapphire Fabry–Pérot Cavity for Pressure Sensing. IEEE Photonics Technol. Lett..

[9] Christmas S.P., Jackson D.A., Henderson P.J., Zhang L., Bennion I., Dalton T., Butler P., Whelan M., Kenny R. (2000). High-resolution vibration measurements using wavelength-demultiplexed fibre Fabry-Perot sensors. Meas. Sci. Technol..

[10] Jiang M., Gerhard E. (2001). A simple strain sensor using a thin film as a low-finesse fiber-optic Fabry–Perot interferometer. Sens. Actuators Phys..

[11] Majchrowicz D., Hirsch M., Wierzba P., Bechelany M., Viter R., Jędrzejewska-Szczerska M. (2016). Application of thin ZnO ALD layers in fiber-optic Fabry-Pérot sensing interferometers. Sensors.

[12] Hirsch M., Majchrowicz D., Wierzba P., Weber M., Bechelany M., Jędrzejewska-Szczerska M. (2017). Low-Coherence Interferometric Fiber-Optic Sensors with Potential Applications as Biosensors. Sensors.

[13] Wang J., Xiao H., Deng I., May R., Wang A. Self-calibrated interferometric/intensity-based (SCIIB) optical fiber pressure sensor. Proceedings of the Process Monitoring with Optical Fibers and Harsh Environment Sensors.

[14] Jiang Y. (2008). Fourier transform white-light interferometry for the measurement of fiber-optic extrinsic Fabry–PÉrot interferometric sensors. IEEE Photonics Technol. Lett..

[15] Wang Z., Jiang Y., Ding W., Gao R. (2013). Fourier transform white-light interferometry based on nonlinear wavelength sampling. Opt. Eng..

[16] Pisani M., Zucco M. (2009). Compact imaging spectrometer combining Fourier transform spectroscopy with a Fabry-Perot interferometer. Opt. Exp..

[17] Vo Q., Fang F., Zhang X., Gao H. (2017). Surface recovery algorithm in white light interferometry based on combined white light phase shifting and fast Fourier transform algorithms. Appl. Opt..

[18] Zhou X., Yu Q. (2011). Wide-range displacement sensor based on fiber-optic Fabry–Perot interferometer for subnanometer measurement. IEEE Sens. J..

[19] Wang Z., Jiang Y., Ding W., Gao R. A cross-correlation based fiber optic white-light interferometry with wavelet transform denoising. Proceedings of the 4th Asia Pacific Optical Sensors Conference.

[20] Xie J., Wang F., Yao P., Wang J., Hu Z., Hu Y. (2015). High resolution signal-processing method for extrinsic Fabry–Perot interferometric sensors. Opt. Fiber Technol..

[21] Bhatia V., Jones M.E., Grace J.L., Murphy K.A., Claus R.O., Greene J.A., Tran T.A. Applications of “absolute” fiber optic sensors to smart materials and structures. Proceedings of the Tenth International Conference on Optical Fibre Sensors.

[22] Leng J.S., Asundi A. (2002). Real-time cure monitoring of smart composite materials using extrinsic Fabry-Perot interferometer and fiber Bragg grating sensors. Smart Mater. Struct..

[23] Jiang Y., Tang C. (2008). High-finesse micro-lens fiber-optic extrinsic Fabry–Perot interferometric sensors. Smart Mater. Struct..

[24] Jiang Y. (2008). High-resolution interrogation technique for fiber optic extrinsic Fabry-Perot interferometric sensors by the peak-to-peak method. Appl. Opt..

[25] Zhang T.Y., Wang W., Chen H.B., Zhang X.X., Ma Z.B., Lv W.T. (2019). Extrinsic Fabry–Perot interferometric cavity-based fiber-optic spectrum equalization filter for the Gaussian spectrum of superluminescent diodes. Appl. Opt..

[26] Ismailoglu N., Yalcin T. Low-power design of a digital FM demodulator based on zero-cross detection at IF. Proceedings of the Gateway to 21st Century Communications Village. VTC 1999-Fall. IEEE VTS 50th Vehicular Technology Conference (Cat. No. 99CH36324).

